# Validation Analysis of a Geriatric Dehydration Screening Tool in Community-Dwelling and Institutionalized Elderly People

**DOI:** 10.3390/ijerph120302700

**Published:** 2015-03-02

**Authors:** Susana Rodrigues, Joana Silva, Milton Severo, Cátia Inácio, Patrícia Padrão, Carla Lopes, Joana Carvalho, Isabel do Carmo, Pedro Moreira

**Affiliations:** 1 Faculty of Medicine, University of Lisbon, Av. Prof. Egas Moniz, Lisbon 1649-028, Portugal; E-Mails: susanarodrigues.nut@gmail.com (S.R.); isabel.carmo72@gmail.com (I.C.); 2 Faculty of Nutrition and Food Sciences, University of Porto, Rua Dr. Roberto Frias, Porto 4200-465, Portugal; E-Mails: joanafcosta.silva@gmail.com (J.S.); patriciapadrao@fcna.up.pt (P.P.); 3 Faculty of Sciences, University of Porto, Rua do Campo Alegre, Porto 4169-007, Portugal; 4 Faculty of Medicine, University of Porto, Al. Prof. Hernâni Monteiro, Porto 4200-319, Portugal; E-Mails: milton@med.up.pt (M.S.); carlal@med.up.pt (C.L.); 5 Institute of Public Health, University of Porto, Rua das Taipas, 135, Porto 4050-600, Portugal; 6 Santa Casa da Misericórdia de Santarém, Largo Cândido dos Reis—Apartado 23, Santarém 2001-901, Portugal; E-Mail: catia.inacio@scms.pt; 7 Research Center of Physical Activity, Health and Leisure, Faculty of Sports, University of Porto, Rua Dr. Plácido Costa, 91, Porto 4200-450, Portugal; E-Mail: jcarvalho@fade.up.pt

**Keywords:** dehydration screening tool, hydration, elderly, institutionalized, community-dwelling

## Abstract

Dehydration is common among elderly people. The aim of this study was to perform validation analysis of a geriatric dehydration-screening tool (DST) in the assessment of hydration status in elderly people. This tool was based on the DST proposed by Vivanti *et al.*, which is composed by 11 items (four physical signs of dehydration and seven questions about thirst sensation, pain and mobility), with four questions extra about drinking habits. The resulting questionnaire was evaluated in a convenience sample comprising institutionalized (*n* = 29) and community-dwelling (*n* = 74) elderly people. Urinary parameters were assessed (24-h urine osmolality and volume) and free water reserve (FWR) was calculated. Exploratory factor analysis was used to evaluate the scale’s dimensionality and Cronbach’s alpha was used to measure the reliability of each subscale. Construct’s validity was tested using linear regression to estimate the association between scores in each dimension and urinary parameters. Two factors emerged from factor analysis, which were named “Hydration Score” and “Pain Score”, and both subscales showed acceptable reliabilities. The “Hydration Score” was negatively associated with 24-h urine osmolality in community-dwelling; and the “Pain Score” was negatively associated with 24-h urine osmolality, and positively associated with 24-h urine volume and FWR in institutionalized elderly people.

## 1. Introduction

Dehydration is a common condition among elderly people being considered a precipitating factor for a number of acute medical conditions. Although it is not clear if there is any causal relationship, there is an association between a low usual fluid intake and some chronic diseases, including urolithiasis, constipation, asthma, cardiovascular disease, diabetic hyperglycemia, and some cancers [1]. In elderly people, dehydration may also precipitate impaired cognitive function, falling, renal failure, pressure ulcers, and poor control of hyperglycemia in diabetes [2]. The risk of infection in elderly individuals has also been linked to poor fluid status [1].

In residential care, dehydration has been proposed as an indicator of the quality of care [3]. Considering that prevention of dehydration may improve health, functional status and quality of life [4], the development of a practical dehydration screening method that could be applied by caregivers without specific clinical skills, would be important to identify older people at risk and prioritize resources for diagnosis and treatment [5].

The most accepted method for dehydration’s diagnosis is the assessment of body fluid loss by weight change, but this has limitations such as the need of two measurements taken over two time points, which precludes the immediate assessment. Other methods have been used to characterize hydration status. Despite plasma osmolality is considered a good marker to assess acute hydration changes, it is not adequate to assess chronic hydration status because it changes constantly [6]. Bioelectrical impedance analysis enables the determination of total body water content and is an easy and quick method, but it is affected by numerous conditions [7] and, despite that it may be useful to assess and monitor hydration status in long-term care facilities [8], its use in epidemiological studies is still under debate [6]. Urinary parameters have been described as valid indices of hydration status in large populations studies [6,7] and the physiologically based concept called “Free Water Reserve” has been used to describe the 24-h hydration status [9]. Collection of urine samples is noninvasive and cheap, and urinary marker measurements can be carried out quickly and do not require high technical expertise [6]. However, these methods are a poor practical option in hydration assessment amongst older people at an individual level, due to the costs associated with urine analysis and also because mobility impairment, which is common in this age group, also limits the collection of complete urine samples. Given these limitations, other means to assess dehydration in non-clinical settings are required and physical signs are mentioned as a preferable assessment strategy [3]. Vivanti *et al.* (2010) [5] studied a large number of potential screening parameters to assess hydration status in hospitalized elderly people, resulting in a dehydration screening tool (DST) composed by the most promising 11 items, which were tested in an independent sample. Their results showed that tongue dryness was the most strongly associated with poor hydration status in hospitalized elderly people.

Considering the importance of preventing dehydration among elderly people, the existence of a screening tool to assess hydration status in a non-clinical setting would be very helpful to enable early intervention. Thus, the main aim of this study was to perform validation analysis of a new geriatric DST in the assessment of hydration status in community-dwelling and institutionalized elderly people.

## 2. Experimental Section

### 2.1. Study Design and Sample

An observational, analytic, cross-sectional design was used in the present study. General data collection was undertaken between November 2012 and June 2013. All subjects gave their informed consent for inclusion before they participated in the study. The study was conducted in accordance with the Declaration of Helsinki, and the protocol was approved by the Ethics Committee of Hospital Center Lisbon-North/Faculty of Medicine, University of Lisbon, and University of Porto (Authorization n° 1OICEIIP12O12).

This study enrolled two Portuguese elderly populations: community-dwelling (those who live in their own homes) and institutionalized elderly people (living in long-term care residences or attending day centers), selected on the basis of convenience sampling. The first group involved elderly adults participating in a Physical Activity (PA) Program from the Faculty of Sport of University of Porto, Porto. All these community-dwelling individuals were responsible for preparing their meals. The institutionalized individuals were recruited in five elderly care centers from the same geographical area. The institutions provided all the daily diet of the study participants.

Sixty-year or older subjects attending the physical activity class during our first visit to the Faculty of Sport of University of Porto and who were at the target elderly care centers were invited to enroll this study. The institutionalized individuals were included if they were institutionalized for at least 30 days.

Details about the study were verbally explained to the participants, including why the research was being conducted, what the study involved, the methods and procedures employed, and the contact details for any necessary support. Subjects were also informed that participation was voluntary and that they were able to withdraw at any time. After the explanation, written consent was obtained from all participants.

Exclusion criteria were the administration of diuretics, due to their impact on urine output [10,11]; the presence of cognitive impairment (assessed by MMSE score) [12,13], due to the inability to complete the interviews; and incomplete 24-h urine samples, which were assessed for completeness using creatinine excretion in relation to weight (*i.e.*, creatinine excretion = creatinine (mg/day)/body weight (kg)). Creatinine coefficients of 14.4 to 33.6 in men and 10.8 to 25.2 in women were classified as indicating an acceptable 24-h urine collection [14]. The cutoff values of MMSE score for Portuguese people were used to exclude subjects with cognitive impairment: less than 22 points (0–2 years of schooling), 24 points (3–6 years of schooling) and 27 points (7 or more years of schooling) were excluded due to cognitive deficit [12,13].

**Figure 1:**
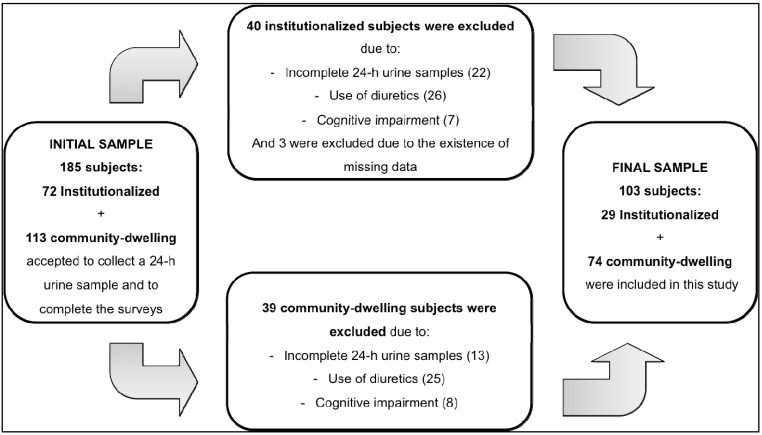
Flow chart of study sample and exclusion criteria.

A total of 113 community-dwelling and 72 institutionalized elderly subjects were screened, although 39 and 43, respectively, community-dwelling and institutionalized, were excluded according to exclusion criteria. Thus, the final sample consisted of 103 elderly individuals (28.2% institutionalized) (Figure 1), who completed the DST and the urine collection.

### 2.2. Data Collection

A trained interviewer collected information on socio-demographic (age, sex, and education level) and clinical data (use of drugs and previously diagnosed health conditions). For the analysis, subjects were classified into two age groups (60–79 years and 80 years or over), according to Vivanti *et al.* (2007) [15]. Regarding education, subjects were grouped into two categories according education attainment: no schooling (including individuals with less than primary education level) or at least primary education level.

Body weight and height were assessed according to standard procedures [16]. Body mass index (BMI) was estimated, and participants were classified according to WHO BMI reference values [17]; for the analysis, BMI was recoded into the following two categories: ≤27.0 or >27.0 kg/m^2^, according to the conclusion of the WHO report that the most favorable BMI for adults 60 years or older are 21.0–27.0 kg/m^2^ for men and 23.0–27.0 kg/m^2^ for women [18].

Considering the impact of alcohol in hydration status [19,20], a 24-h dietary recall was performed parallel to the urine collection. The recalls were obtained through face-to-face interviews and complementary use of photographic models [21]. Data from dietary recalls were processed using the software Food Processor Plus^®^ 10.0 (ESHA Research, Salem, OR, USA). Nutritional data about Portuguese foods and beverages were obtained from the Portuguese Food Composition Table [22] and added to the software’s basis, and alcohol beverages and alcohol (ethanol) daily intake were estimated.

### 2.3. Urine Collection and Hydration Markers

Participants received oral and written explanations on the urine collection, being instructed to collect a 24-h urine sample. All subjects were instructed to avoid several common errors that lead to omission of a small part of the specimen, to maintain their eating and drinking habits during the urine collection period, and to keep the urine refrigerated. At the end of the collection, completeness was assessed through a non-judgmental interview.

Urine samples were analyzed for 24-h creatinine (mg/day), and urine hydration markers, namely 24-h urine volume (mL), and 24-h urine osmolality (UOSM, mOsm/kg) [6,23]. All analyzes were performed in a certified laboratory. Renal solute excretion was calculated from 24-h urine volume and osmolality. The results were stratified by sex, due to the recognized variability between men and women in urinary parameters [9].

Hydration status was also characterized by free water reserve (FWR), which was calculated from measured urine volume (mL/24-h) minus the obligatory urine volume (mL/24-h)] [9]. Obligatory volume is the ideal urine volume to excrete the actual 24-h urine solutes at the mean—2 standard deviation value of maximum UOSM (subjects ≥ 20 years = 830 − 3.4 (age − 20) mOsm/kg) [24,25]. Subjects were classified as hypohydrated if FWR was negative and euhydrated if it was positive [9,24,25].

### 2.4. Dehydration Screening Tool

The geriatric DST in this study was developed based on the Vivanti *et al.* (2010) [5] previous questionnaire and a set of four questions regarding the drinking habits and the level of carefulness about hydration status was added.

The tool developed by Vivanti *et al.* (2010) [5] includes four physical signs of dehydration (drop on systolic blood pressure, tongue dryness, skin turgor and body weight) and seven items about thirst sensation, pain and mobility, which were found to be associated with hydration status in hospitalized elderly people by Vivanti *et al.* (2010) [5]. In the study of the potential factors associated with dehydration in older adults, Vivanti (2007) [15] included questions about pain and mobility due to the reported evidence in the literature of a possible association with dehydration. The results of that study showed that the dehydrated individuals were more likely to indicate they never had difficulties with pain and mobility, when compared to the well hydrated.

The DST, originally written in English, was translated into Portuguese by three health researchers. This Portuguese version was back-translated into English by an independent native English speaker and was compared with the original version to ensure equivalence between the two versions. Discrepancies were decided by unanimous agreement. Therefore, the instrument was tested in small pilot sample of elderly subjects (*n* = 10) to evaluate its comprehension.

This instrument was applied in both elderly groups, shortly after the collection of urine samples. It required the measurement of blood pressure, which was performed using a standardized automated sphygmomanometer (OMRON^®^ M6 Comfort HEM-7000-E). Systolic blood pressure was measured in the sitting position and after two minutes while standing. The drop in systolic blood pressure on standing was considered to be significant if it was at least 20 mmHg. Skin turgor was assessed pinching the skin on the dorsum of the hand and recording the seconds elapsed for tissue fold to return to normal. If it had elapsed two seconds or more, skin was considered to present a poor turgor.

A set of four questions regarding the habits of drinking water or other beverages at meals (“Do you like to drink water?”; “Do you usually drink at meals?”; and “If you have several beverages available, do you usually choose water?”), and the level of carefulness about hydration status (“Are you concerned about being well hydrated?”), was added to the original scale of eleven items from Vivanti *et al.* [5] and the resulting final scale comprised fifteen dichotomous items (yes or no). The responses to the items were codified using the criteria of Vivanti (2007) [15]: 1 point was assigned to items that described the absence of a characteristic or symptom related to dehydration (and no points if the characteristic or symptom was present). Only item 3, which was initially codified in the opposite direction of the other items according to the results of Vivanti (2007) [15], was recodified in our analysis because the answers were in the same direction of the others. The items whose factor loadings were above 0.4 were selected to estimate a total score for each subscale after factorial analysis [26].

### 2.5. Statistical Analysis

Descriptive statistics were used to present demographic characteristics. Continuous variables (age, weight, height and BMI) were described as mean and standard deviation, and categorical variables (sex and education level) were summarized as counts and percentages. Shapiro-Wilk test was performed to test variables for normality. Continuous variables were compared between the institutionalized and non-institutionalized samples using t-test for independent samples, when data were normally distributed, and Mann-Whitney U test, if data were not normally distributed. Categorical dichotomous variables were tested using the Chi-square test.

Exploratory factor analysis for dichotomous variables (latent trait models) was performed to make the decision about the items that significantly contributed to the scale. Extraction method for exploratory factor analysis was the robust weighted least squares. The factors are the correlated under oblique geomin rotation. The items whose factor loadings were above 0.4 were selected and the reliability of the resulting construct was assessed using Cronbach’s Alpha, in the overall sample. Reliabilities of 0.5 are satisfactory for short dichotomous scales (10–15 items) [27].

Linear regression was used to estimate the association between the construct and the urinary parameters, in institutionalized and community-dwelling elderly people. Analysis included a crude model—Model 1, and an adjusted model—Model 2, adjusted for age and sex.

Statistical analysis was conducted using SPSS Statistical Package^®^ 21.0 (IBM Corporation, 2012, Armonk, NY, USA). Factorial analysis for dichotomous variables was performed using the software R 3.0.1, and specifically the ltm package. A *p*-value < 0.05 was considered to indicate statistical significance.

## 3. Results and Discussion

This study enrolled 103 elderly individuals (28.2% institutionalized) aged 60–94 years old (73.7 ± 8.4 years). Socio-demographic data presented in Table 1 showed that the institutionalized elderly individuals were significantly older than the community-dwellers (82.5 ± 7.2 *vs.* 70.2 ± 6.0 years, *p* < 0.001). Women were in higher proportion both in institutionalized (55.2%) and community-dwelling elderly subjects (62.2%). Community-dwelling participants reported a higher education attainment, being 41.9% classified with middle school or higher level, compared to 30.0% in the institutionalized group.

**Table 1:** Participants characteristics.

Characteristics	Institutionalized (*n* = 29)	Community-Dwelling (*n* = 74)	*p*-value	Total (*n* = 103)
Age (years), mean ± SD	82.5 ± 7.2	70.2 ± 6.0	<0.001 ^*^	73.7 ± 8.4
60–79 years	8 (27.6%)	67 (90.5%)	<0.001 ^‡^	75 (72.8%)
≥80 years	21 (72.4%)	7 (9.5%)	--	28 (27.2%)
**Sex, *n* (%)**				
Male	13 (44.8%)	28 (37.8%)	0.515 ^‡^	41 (39.8%)
Female	16 (55.2%)	46 (62.2%)		62 (60.2%)
Score MMSE, median (range)	24 (22, 29)	29 (22, 30)	<0.001 ^*^	28 (22, 30)
**Education level, *n* (%)**				
No schooling	14 (48.3%)	14 (18.9%)	0.002 ^‡^	28 (27.2%)
Primary school	15 (51.7%)	29 (39.2%)	--	44 (42.7%)
Middle school	0 (0.0%)	19 (25.7%)	--	19 (18.4%)
Secondary school	0 (0.0%)	6 (8.1%)	--	6 (5.8%)
Higher school	0 (0.0%)	6 (8.1%)	--	6 (5.8%)
BMI (kg/m^2^), mean ± SD	27.8 ± 4.0	27.1 ± 3.7	0.410 ^†^	27.3 ± 3.8
Underweight (<18.5 kg/m^2^)	1 (3.4%)	2 (2.7%)	0.644 ^‡^	3 (2.9%)
Normal weight (18.5–24.9 kg/m^2^)	4 (13.8%)	18 (24.3%)	--	22 (21.4%)
Overweight (25.0–29.9 kg/m^2^)	15 (51.7%)	37 (50.0%)	--	52 (50.5%)
Obesity (≥30 kg/m^2^)	9 (31.0%)	17 (23.0%)	--	26 (25.2%)
Total number of drugs, median (range)	6 (1, 14)	3 (0, 8)	< 0.001 ^*^	3 (0, 14)
**Health condition, *n* (%)**				
Hypertension	23 (79.3%)	39 (52.7%)	0.010 ^‡^	62 (60.2%)
Dyslipidemias	10 (34.5%)	37 (50.5%)	0.123 ^‡^	47 (45.6%)
Arthritis	4 (13.8%)	3 (4.1%)	0.075 ^‡^	7 (6.8%)
Diabetes mellitus	9 (31.0%)	10 (13.5%)	0.037 ^‡^	19 (18.4%)
Renal impairment	0 (0.0%)	4 (5.4%)	0.206 ^‡^	4 (3.9%)
Alcoholic beverages consumption, *n* (%)	4 (13.8%)	28 (37.8%)	0.014 ^‡^	32 (31.1%)
24-h wine consumption (mL), median (range) ^§^	175 (100, 300)	225 (40, 1250)	0.931 ^*^	200 (40, 1250)
24-h alcohol intake (g), median (range) ^§^	16.1 (9.2, 27.6)	20.7 (3.7, 115.0)	0.934 ^*^	18.4 (3.7, 115.0)

^*^ Mann-Whitney U test; **^†^** T-test for independent samples; **^‡^** Chi-square test; **^§^** 24-h volume of alcoholic beverages consumed and alcohol intake among drinkers; BMI—Body mass index.

Anthropometric evaluation showed a similar distribution of BMI categories amongst the institutionalized and the community-dwelling elderly group. The analysis of health condition data (Table 1) showed that the institutionalized elderly people took a significantly higher number of prescription drugs than the community-dwelling subjects (6.6 ± 2.9 *vs.* 3.0 ± 1.9, *p* < 0.001) and showed a higher prevalence of hypertension (79.3% *vs.* 52.7%, *p* = 0.010) and diabetes mellitus (31.0% *vs.* 13.5%, *p* = 0.037).

Significantly more community-dwellers consumed alcoholic beverages than the institutionalized elders (13.8% *vs.* 37.8%, *p* = 0.014). The only beverage consumed was wine and the mean 24-h consumption was not different between institutionalized and community-dwelling elderly people who commonly consumed alcoholic beverages.

Analysis of urine biomarkers is presented in Table 2. The 24-h UOSM and the 24-h urine volume did not differ significantly between institutionalized and community-dwelling elderly women. However, community-dwelling men showed a significantly higher 24-h urine volume (1835 mL *vs.* 1750 mL, *p* = 0.049 and a significantly lower 24-h UOSM (414 mOsm/kg *vs.* 564mOsm/kg, *p* = 0.015) than the institutionalized men. FWR was also significantly higher in community-dwelling than in institutionalized elderly men (780 mL/24h *vs.* 134 mL/24h, *p* < 0.001). Community-dwelling and institutionalized participants showed similar renal solute excretion.

**Table 2:** Urinary parameters of institutionalized and community-dwelling elderly people by sex.

Urinary Parameters	Institutionalized (*n* = 29)	Community-dwelling (*n* = 74)	*p*-value	Total (*n* = 103)
**Males**				
24-h urine volume, mL	1750 (800, 2100)	1835 (650, 3300)	0.049 ^*^	1790 (650, 3300)
24-h UOSM, mOsm/kg	564 (391, 781)	414 (225, 768)	0.015 ^†^	462 (225, 781)
Renal solute excretion, mOsm/24-h	880 (586, 1562)	801 (499, 1262)	0.195 ^*^	865 (499, 1562)
Free Water Reserve, mL/24-h	134 (−537, 595)	780 (−157, 1823)	<0.001 ^*^	461 (−537, 1823)
**Females**				
24-h urine volume, *mL*	1450 (750, 2800)	1750 (760, 3450)	0.927 ^*^	1625 (750, 3450)
24-h UOSM, mOsm/kg	380 (272, 620)	377 (84, 799)	0.129 ^*^	377 (84, 799)
Renal solute excretion, mOsm/24-h	623 (257, 1056)	619 (260, 1073)	0.300 ^*^	621 (257, 1073)
Free Water Reserve, mL/24-h	621 (−1, 1184)	720 (−207, 2706)	0.147 ^*^	706 (−207, 2706)

Values are expressed as median (range); ^*^ *t*-test for independent samples; **^†^** Mann-Whitney U test; UOSM—Urine osmolality.

Factor analysis of the fifteen items led to the exclusion of four items related to physical signs of dehydration (drop on systolic blood pressure on standing, tongue dryness, reduced skin turgor and reduced body weight), which presented very low factorial loadings. The factor analysis of the resulting eleven items (Table 3), showed the existence of two distinct factors, which were named “Pain Score” and “Hydration Score”.

**Table 3:** Factor analysis of the items considered in the Dehydration Screening Tool (DST).

Dehydration Screening Tool (DST) Items	Pain Score (Factor 1)	Hydration Score (Factor 2)
1—Do you ever feel thirsty?	0.383	**0.539**
2—Did you feel thirsty yesterday?	0.386	**0.504**
3—Do you have difficulty moving your shoulders, arms or hands?	**−0.851**	−0.007
4— In the past 2 weeks, did pain interfere with your daily activities?	**0.987**	0.161
5—In the past 2 weeks did you have problems with pain of any kind?	**0.785**	0.044
6—In the last 2 weeks, did you drop something?	**0.466**	0.037
7—How many times have you had a headache in the past week?	**0.569**	0.278
8—Do you like to drink water?	0.058	**−0.685**
9—Do you usually drink at meals?	0.064	−0.189
10—If you have several beverages available, do you usually choose water?	0.038	**−0.585**
11—Are you concerned about being well hydrated?	0.197	**−0.927**

The “Pain Score” showed a Cronbach’s Alpha of 0.66. The following five items composed this factor: (a) “Do you have difficulty moving your shoulders, arms or hands?”; (b) “In the past 2 weeks, did pain interfere with your daily activities?”; (c) “In the past 2 weeks, did you have problems with pain of any kind?”; (d) “In the past 2 weeks, did you drop something?”; and (e) “How many times have you had a headache in the past week?”.

Regarding the “Hydration Score”, it was composed by five items, all of them being potential indicators of a good hydration status: (a) “Do you ever feel thirsty?”; (b) “Did you feel thirsty yesterday?”; (c) “Do you like to drink water?”; (d) “If you have several beverages available, do you usually choose water?”; and (e) “Are you concerned about being well hydrated?”. This factor also showed a good reliability, since it showed a Cronbach’s Alpha of 0.58. One item was excluded from both scores (“Do you usually drink at meals?”) since it showed a loading below 0.4 for the two factors.

### 3.1. “Pain Score”, “Hydration Score” and Urinary Parameters

The mean total values of each factor (“Hydration Score” and “Pain Score”) are shown in Table 4. Results showed significant differences between institutionalized and community-dwelling elderly, with institutionalized subjects showing higher scores. The older group (≥80 years) also showed higher “Hydration Score” and “Pain Score” than the younger group. Women showed significantly higher “Pain Score” than men (2 *vs.* 1, *p* < 0.001), but there were no significant differences between sexes in what concerns to “Hydration Score”. “Hydration Score” and “Pain Score” were not significantly different between groups with different education levels and BMI.

The associations between urinary parameters and both the “Pain Score” and the “Hydration Score” are shown in Table 5. Results are presented in institutionalized and community-dwelling elderly, considering that significant differences were found in urinary parameters between these groups.

In the total sample, none of the urinary parameters was significantly associated with “Pain Score” or “Hydration Score”. In the institutionalized elderly group, 24-h urine volume was significantly and positively associated with “Pain Score” (*r*^2^ = 0.17, *p* = 0.026), while 24-h UOSM was significantly and negatively associated with “Pain Score” (*r*^2^ = 0.25, *p* = 0.006). FWR was also positively associated with “Pain Score” in institutionalized elderly people (*r*^2^ = 0.32, *p* = 0.002). When adjusted for age and gender (Model 2), the associations remained significant for 24-h urine volume (*r*^2^ = 0.24, *p* = 0.011) and FWR (*r*^2^ = 0.46, *p* = 0.012), and lost the significance in what concerns to 24-h UOSM (*r*^2^ = 0.46, *p* = 0.051).

**Table 4:** “Hydration Score” and “Pain Score” according to socio-demographic variables.

Socio-Demographic Variables	*n*	Hydration Score	*p*-value ^*^	Pain Score	*p*-value ^*^
**Settings**					
*Institutionalized*	29	3 (0, 5)	0.013	3 (0, 5)	0.003
*Community-dwelling*	74	2 (0, 5)		1 (0, 5)	
**Age**					
*60–79 years*	75	2 (0, 5)	0.010	1 (0, 5)	0.016
*≥ 80 years*	28	3 (0, 5)		3 (0, 5)	
**Sex**					
*Male*	41	2 (0, 5)	0.989	1 (0, 4)	<0.001
*Female*	62	2 (0, 5)		2 (0, 5)	
Education level					
*No schooling*	28	3 (0, 5)	0.115	2 (0, 5)	0.145
*At least primary level*	75	2 (0, 5)		1 (0, 5)	
**BMI**					
*≤27.0 kg/m^2^*	52	2 (0, 5)	0.793	1 (0, 5)	0.774
*>27.0 kg/m^2^*	51	2 (0, 5)		1 (0, 5)	

Values are expressed as median (range); ^*^ Mann-Whitney U test; BMI—Body mass index.

**Table 5:** Relation of “Pain Score” and “Hydration Score” with urinary parameters.

**24-h Urine Volume (mL)**						
	**Total Sample (*n* = 103)**		**Institutionalized (*n* = 29)**		**Community-Dwelling (*n* = 74)**	
	B (95% CI)	*p* value	B (95% CI)	*p* value	B (95% CI)	*p* value
Model 1 ^*^						
Pain Score	−5.0 (−88.1, 78.2)	0.906	124.0 (15.7, 232.3)	0.026	−32.7 (−146.7, 81.2)	0.569
Hydration Score	61.9 (−39.1, 162.8)	0.227	69.4 (−60.3, 199.1)	0.282	123.8 (−20.9, 268.4)	0.092
**Model 2 ^†^**						
Pain Score	25.1 (−65.9, 116.1)	0.060	155.6 (39.6, 271.6)	0.011	−11.4 (−138.4, 115.6)	0.858
Hydration Score	69.5 (−31.5, −170.5)	0.135	75.9 (−62.5, 214.2)	0.270	119.8 (−26.7, 266.3)	0.107
**24-h Urine Osmolality (mOsm/kg)**						
	**Total Sample (*n* = 103)**		**Institutionalized (*n* = 29)**		**Community-dwelling (*n* = 74)**	
	B (95% CI)	*p* value	B (95% CI)	*p* value	B (95% CI)	*p* value
Model 1 ^*^						
Pain Score	−13.0 (−33.4, 7.4)	0.208	−45.9 (−77.2, −14.6)	0.006	−6.0 (−32.8, 20.8)	0.658
Hydration Score	−19.3 (−44.1, 5.5)	0.126	−15.4 (−55.3, 24.4)	0.435	−37.4 (−70.9, −3.8)	0.029
**Model 2 ^†^**						
Pain Score	−1.1 (−22.9, 20.7)	0.921	−29.6 (−59.4, 0.1)	0.051	1.3 (−27.2, 29.7)	0.929
Hydration Score	−19.6 (−43.7, 4.5)	0.109	−10.3 (−44.5, 23.8)	0.539	−40.9 (−72.9, −9.0)	0.013
**Free Water Reserve (mL/24-h)**						
	**Total Sample (*n* = 103)**		**Institutionalized (*n* = 29)**		**Community-dwelling (*n* = 74)**	
	B (95% CI)	*p* value	B (95% CI)	*p* value	B (95% CI)	*p* value
Model 1 ^*^						
Pain Score	17.4 (−62.8, 97.7)	0.668	144.6 (60.4, 228.8)	0.002	11.2 (−98.0, 120.5)	0.838
Hydration Score	67.0 (−30.3, 164.4)	0.175	72.2 (−37.7, 182.0)	0.189	154.7 (18.3, 291.1)	0.027
**Model 2 ^†^**						
Pain Score	7.5 (−79.0, 93.9)	0.864	110.1 (26.6, 193.6)	0.012	−1.3 (−122.9, 120.3)	0.983
Hydration Score	79.6 (−15.8, 175.0)	0.101	45.8 (−54.1, 145.7)	0.354	160.8 (23.1, 298.4)	0.023

^*^ Unadjusted model; **^†^** Adjusted for age (years) and gender; CI—Confidence interval.

In community-dwelling subjects, none of the urinary indicators of hydration status was significantly associated with the “Pain Score”. However, in this group, 24-h UOSM was significantly associated with the “Hydration Score”, both in the unadjusted (*r*^2^ = 0.06, *p* = 0.029) and in the adjusted model (*r*^2^ = 0.18, *p* = 0.013). The 24-h urine volume did not show significant association with “Hydration Score” (*p* = 0.107) among community-dwelling elderly people, but FWR was positively associated with “Hydration Score” both in the unadjusted (*r*^2^ = 0.07, *p* = 0.027) and in the adjusted (*r*^2^ = 0.08, *p* = 0.023) models.

Table 6 shows that only six institutionalized and six community-dwelling elderly subjects were hypohydrated and that there were no significant differences in “Pain Score” and “Hydration Score” between euhydrated and hypohydrated subjects. However, among institutionalized subjects, those with “Pain Score” <4 showed a significantly lower FWR, when compared with those with “Pain Score” ≥4 (244.2 ± 358.1 *vs.* 765.6 ± 343.8 mL/24-h, *p* = 0.002). Moreover, community-dwelling subjects with “Hydration Score” <3 showed a significantly lower FWR when compared with those with “Hydration Score ≥ 3 (1004.9 ± 604.4 *vs.* 673.9 ± 613.6, *p* = 0.031).

**Table 6:** “Pain Score” and “Hydration Score” in euhydrated and hypohydrated elderly people.

Scores	Euhydrated	Hypohydrated	*p*-value
**Total Sample**, *n*	91	12	--
Pain Score	1 (0, 5)	1 (0,3)	0.280 ^†^
Hydration Score	2 (0, 5)	2 (0, 5)	0.290 ^†^
**Institutionalized**, *n*	23	6	--
Pain Score	3 (0, 5)	1.5 (0, 3)	0.065 ^*^
Hydration Score	3 (0, 5)	2.5 (0, 5)	0.670 ^*^
**Community-dwelling**, *n*	68	6	--
Pain Score	1 (0, 5)	1 (0, 3)	0.623 ^†^
Hydration Score	2 (0, 5)	1.5 (1, 2)	0.065 ^†^

Values are expressed as median (range); ^*^ *t*-test for independent samples; **^†^** Mann-Whitney U test.

### 3.2. Discussion

The main achievement of this study was the contribution to the development of a new DST that could be used in the identification of elders at greater risk of dehydration both in institutionalized and in community-dwelling populations. Two factors emerged in the validation analysis, which were called “Hydration Score”, consisting of five questions about thirst sensation and preferences related to fluid consumption, and “Pain Score”, consisting of five items about mobility and pain. These 10 questions were considered in the new DST. Although Cronbach’s alpha should be higher than 0.70, considering the relatively small number of items in each subscale and the sample size, the reliability of both scores was considered to be acceptable because, according to Kehoe (1995) [27], reliabilities of 0.5 are satisfactory for short dichotomous scales. The reliability and validity were tested in the overall sample and not among subpopulations since our main objective was to create and test a tool able to measure hydration status in elderly individuals regardless their age, other socio-demographic characteristics or living conditions. In our view, the dehydration construct does not vary between elderly groups, while the intensity of the dehydration is expected to be different among subgroups. Therefore we opted to include a relatively heterogeneous sample of 103 elderly individuals, aged 60–94 years, with approximately one third institutionalized. However, the resulting constructs did not show association with urinary parameters in the total sample. Considering the existence of two different sub-populations, analysis was performed to ascertain the existence of associations in each sub-sample, adjusting for age and gender to eliminate the possible confound effect of these variables.

The two resulting scores showed different associations with urinary parameters in both elderly populations. The “Hydration Score” did not show any association with urinary parameters in institutionalized elderly people, while in the community-dwellers, it showed a negative association with the 24-h urine osmolality (18% of the variance being explained by the adjusted model) and a positive weak association with FWR (the adjusted model explained only 8% of the variance), which suggests that “Hydration Score” may have an association with hydration status in community-dwellers, but not in institutionalized elderly people. The questions composing this score are mainly related to water intake and do not consider the consumption of other beverages, particularly the alcoholic beverages, which were consumed by 37.8% of community-dwellers (significantly more than in institutionalized elderly people). Despite alcohol consumption might be underestimated by dietary recall, these differences are considerable because drinks containing higher amounts of alcohol contribute less to improve hydration status [19], but their consumption increases diuresis [20], and this may be on the basis of the lack of association between the “Hydration Score” and the 24-h urine volume in community-dwellers. Moreover, considering that institutionalized elders are significantly older, the first two items about thirst composing the “Hydration Score” may contribute for the difference between community-dwelling and institutionalized elderly people. Thirst sensation had previously shown to be a bad predictor of hydration status among elderly people [15,28,29], due to the recognized decline in thirst sensation with aging [28,30,31,32]. In addition, the items “Do you ever feel thirsty?” and “Did you feel thirsty yesterday?” can be interpreted in two different forms: on one hand, elderly individuals who respond “yes” to those questions may drink more to compensate thirst sensation and consequently be better hydrated or, on the other hand, they can feel thirsty due to a poorer hydration status. It is difficult to establish a direct relationship between thirst and hydration status [33]. These results suggest that “Hydration Score” may be useful in the assessment of hydration status in community-dwelling, but not in institutionalized elderly people.

The “Pain Score” showed an association with hydration status in institutionalized elderly people, *i.e.*, individuals with a higher “Pain Score” (reflecting more difficulties with pain and mobility) were more likely to show a better hydration status, characterized by a higher 24-h urine volume, a lower 24-h urine osmolality and a higher FWR. These associations were independent of age and sex, except in what concerns to the 24-h urine osmolality, which lost the statistical significance in the adjusted model (*p* = 0.051). In addition to age and gender, Vivanti (2007) [15] had described that body mass index might confound the hydration status. Other authors have argued that hydration needs for elderly people should take in account their body weight [34]. However, in this study, despite BMI was initially considered to be a confounding variable, this adjustment was disregarded because there were no significant differences in urinary parameters between individuals with different BMI’s. The adjusted model of “Pain Score” explained 46% of the variance of FWR, and 24% of the variance of 24-h urine volume in institutionalized elderly individuals.

Our results regarding “Pain Score” are in agreement with the findings of Vivanti (2007) [15], who found that the individuals clinically assessed as dehydrated indicated no headaches during the previous week, fewer problems with pain of any kind in the past two weeks and less often reported pain interfering with daily activities. However, there are no other evidences supporting this relationship and the mechanisms behind them remain unknown. These findings are controversial because other authors have previously described that mobility impairment was associated with lower fluid intakes [35,36,37,38], but two studies found that nursing home residents with better physical function were at greater risk of dehydration [39,40]. According to Mentes (2006) [41], they “may reflect environmental factors in nursing homes, where caregivers may be more attuned to highly dependent residents who cannot drink independently, leaving those who are able to care for themselves to do so. Such an approach may not work with residents who are physically functional but cognitively impaired.” The association in this study between pain absence and a poor hydration status should be explored, since it could be used in the development of a practical and easy tool to detect institutionalized elders at greater risk and early prevent dehydration.

Nevertheless, the “Pain Score” did not show any association with urinary parameters in the community-dwelling elderly people. This can be explained by the fact that this population, namely the ones enrolled in PA programs, commonly shows fewer problems with pain and mobility than either those institutionalized (in the present study) or hospitalized (in the study by Vivanti [15]). Pain and mobility impairments have been identified as predictor factors for institutionalization [42,43]. According to this, an explanation for the difference in the findings between institutionalized and community-dwelling elderly people may be related to physical activity and this should be explored in next studies.

Overall, the previously described factors, such as advanced age, the low education level, the high number of prescription drugs and others, have been considered to be predictive factors for institutionalization [42] and they can also be putative contributors for the higher risk of dehydration in institutionalized individuals. The results of the present study regarding urinary parameters confirmed that community-dwelling elderly men were better hydrated than the institutionalized men, according the higher 24-h urine volumes, lower 24-h urine osmolality and higher FWR in the community-dwellers. These results are supported by the findings of Leiper *et al.* (2005) [36] who found higher 24-h urine volumes in community-dwellers when compared to institutionalized elderly people. In the study by Leiper and colleagues [36], community-dwellers showed an average 24-h urine volume of 1.7 L and institutionalized elderly people showed mean values of 0.9 to 1.1 L, while in this study the community-dwellers had mean 24-h urine volumes of 1.8 to 2.0 L and the institutionalized individuals of 1.6 L. Other studies have shown high prevalences of inadequate fluid intake [34,38,44,45] and consequently of dehydration among institutionalized elderly people [46,47].

To characterize the hydration status, we used the concept of FWR [9] calculated from the 24-h urine osmolality, which is considered an appropriate marker of hydration status [25], and no significant differences in scores (Pain Score and Hydration Score) were found when comparing hypohydrated and euhydrated subjects. Based in this result, the scores seem to be associated with different levels of euhydration, but do not allow the identification of hypohydrated older people. In an attempt to develop a useful tool for hydration assessment in older people, future studies should include other questions, such as more specific questions about drinking habits, and other physical signs, such as axillary moisture, which has been recently described to be a good marker of hydration status [48,49]. Moreover, next studies should also involve larger samples, particularly including a larger number of subjects in a hypohydrated state.

Almost all of the items from the DST used in the present study were based on previous work developed by Vivanti *et al.* (2010) [5]. The authors applied this instrument in older people admitted in rehabilitative care units of metropolitan hospitals and found that tongue dryness was the parameter that showed the better combination of sensibility and specificity for dehydration detection. They concluded that the assessment of tongue dryness was a simple and quick method, which could be used in individuals with minimal cognitive and physical capacity. However, in the present study our results led to the exclusion of tongue dryness and other three physical signs (postural hypotension, reduced skin turgor, and low body weight). Other authors had previously concluded that physical signs are not useful in the assessment of dehydration in the elderly [31,48,50,51] because they are generally associated with common physiologic age-related changes or with conditions that are more prevalent in elderly individuals, such as polymedication or chronic diseases. Moreover, physical signs of dehydration may be absent or misleading in older people [50]. Recent evidence has supported the use of axillary moisture as a physical sign that is easy to assess hydration status in older people admitted to hospitals [48,49]. However, the validation of its use in community-dwellers and institutionalized elderly people is lacking. The majority of the previous studies regarding physical signs that showed positive outcomes were performed in clinical settings, where the degree of dehydration is expected to be more accentuated. In non-clinical settings, severe dehydration should be prevented and early stages of mild dehydration should be detected. In these stages, physical signs of dehydration may be absent.

## 4. Conclusions

The factor analysis in this study resulted in two subscales, one of them—the “Hydration Score”—Being inversely associated with urine osmolality, but not in institutionalized elderly people, and the other—The “Pain Score”—Showing a significant association with urinary parameters in the institutionalized elderly group. Despite the resulting scales do not allow the identification of hypohydrated elderly people, they showed significant associations with hydration status in the studied populations. This is a promising finding to be considered in future studies oriented to further contribute to the identification of a simple, easy and practical method to identify the elders at greater risk of dehydration in non-clinical settings, particularly in those environments where more objective methods cannot be used, due to low economic resources. Moreover, our findings could also contribute to the identification of a tool that could be easy to apply by anyone, without the need of special skills.

Nevertheless, limitations of the study should be recognized. First, we have a convenience and reduced sample size, which did not allow the extrapolation of the results to all elderly individuals. Second, our findings need replication. Furthermore, the cross-sectional nature of this study precludes us from inferring an underlying relationship between the behaviors measured by DST and urinary parameters. The strengths of this study include an important topic area less studied, particularly in the elderly, and therefore provides information that can be further developed in future studies.
